# A Multi-Disciplinary Review on the Aerobiology of COVID-19 in Dental Settings

**DOI:** 10.3389/fdmed.2021.726395

**Published:** 2021-09-29

**Authors:** Darya Dabiri, Samuel Richard Conti, Niloufar Sadoughi Pour, Andrew Chong, Shaahin Dadjoo, Donya Dabiri, Carol Wiese, Joyce Badal, Margaret Arleen Hoogland, Heather Raquel Conti, Travis Roger Taylor, George Choueiri, Omid Amili

**Affiliations:** 1 Department of Dentistry, Division of Pediatric Dentistry, University of Toledo, Toledo, OH, United States,; 2 Department of Biological Sciences, University of Toledo, Toledo, OH, United States,; 3 Department of Mechanical, Industrial and Manufacturing Engineering (MIME), University of Toledo, Toledo, OH, United States,; 4 Department of Cariology, Restorative Sciences & Endodontics, University of Michigan, Ann Arbor, MI, United States,; 5 Department of Orthodontics and Dentofacial Orthopedics, The Eastman Institute for Oral Health, University of Rochester, Rochester, NY, United States,; 6 Department of Medicine, University of Toledo, Toledo, OH, United States,; 7 Mulford Health Sciences Library, University of Toledo, Toledo, OH, United States,; 8 Department of Medical Microbiology and Immunology, University of Toledo, Toledo, OH, United States

**Keywords:** COVID-19, particle measurement, bioaerosol, dental procedures, particle topography

## Abstract

The COVID-19 pandemic pushed dental health officials around the world to reassess and adjust their existing healthcare practices. As studies on controlled COVID-19 transmission remain challenging, this review focuses on particles that can carry the virus and relevant approaches to mitigate the risk of pathogen transmission in dental offices. This review gives an overview of particles generated in clinical settings and how size influences their distribution, concentration, and generation route. A wide array of pertinent particle characterization and counting methods are reviewed, along with their working range, reliability, and limitations. This is followed by a focus on the effectiveness of personal protective equipment (PPE) and face shields in protecting patients and dentists from aerosols. Direct studies on severe acute respiratory syndrome coronavirus 2 (SARS-CoV-2) are still limited, but the literature supports the use of masks as an important and effective non-pharmaceutical preventive measure that could reduce the risk of contracting a respiratory infection by up to 20%. In addition to discussing about PPE used by most dental care professionals, this review describes other ways by which dental offices can protect patients and dental office personnel, which includes modification of the existing room design, dental equipment, and heating, ventilation, and air conditioning (HVAC) system. More affordable modifications include positioning a high-efficiency particulate air (HEPA) unit within proximity of the patient’s chair or using ultraviolet germicidal irradiation in conjunction with ventilation. Additionally, portable fans could be used to direct airflow in one direction, first through the staff working areas and then through the patient treatment areas, which could decrease the number of airborne particles in dental offices. This review concludes that there is a need for greater awareness amongst dental practitioners about the relationship between particle dynamics and clinical dentistry, and additional research is needed to fill the broad gaps of knowledge in this field.

## INTRODUCTION

Severe acute respiratory syndrome coronavirus 2 (SARS-CoV-2), which is the causative virus of coronavirus disease-2019 (COVID-19), presents challenges greater than that posed by seasonal influenza (R_0_ ~ 1.2), Middle East respiratory syndrome (MERS) (R_0_ ~ 1.4), or severe acute respiratory syndrome (SARS) (R_0_ ~ 3) due to its high reproductive number (R_0_ = 1.4–3.9) (1–3). The most recent global assessment of R_0_ for COVID-19 is 4.08. This high reproductive number contributed to the unprecedented global spread of SARS-CoV-2 (4).

According to a scientific brief published by the WHO on March 29, 2020, the primary transmission mode of SARS-CoV-2 is through respiratory droplets and contact routes (i.e., oral secretions) with a diameter >5 μm (5, 6). These droplets enter the air through speaking, coughing, and sneezing by individuals in close contact with each other (5–8). Short-range inhalation of aerosols can be a route of COVID-19 transmission as with many respiratory pathogens (9). Oral cavity is another important site for SARS-CoV-2 infection (10). The most reported mode of pathogen spread is through respiratory droplets. Individuals that are pre-symptomatic or asymptomatic do not significantly cough or sneeze, yet are responsible for more than 50% of COVID-19 transmission (11), and hence it is important to study the effect of particles expired through normal breathing and oral cavity secretions (10, 12).

Dental practices across the globe closed down for non-emergency dental care during peak COVID-19 periods because of the higher risk of virus transmission during dental appointment due to the close proximity of patient–provider and the use of modern dental tools, such as high-speed handpieces, ultrasonic scalers, air turbines, and air-water syringes, in the presence of contaminated salivary secretions (5, 10, 13–15). It is important to note that dental professionals demonstrated high compliance (72.8%) to CDC guidelines during the COVID-19 pandemic, which has led to very low cases of COVID-19 transmission in dental settings (16). In this review, the authors present studies focused on the transport of particles, the various methods of characterizing the particles, bioaerosols in dentistry, and finally recommendations for reducing the transmission of potentially virus-laden droplets generated during routine dental treatments.

## PARTICLES

To better understand the modifications that could be adopted by dental offices to minimize transmission of SARS-CoV-2, the team elaborates on the definition of particle and droplet, transmission of particles *via* bioaerosols, and the natural production and dissemination of aerosols produced during cleaning or treatment at a dental office.

### Definitions of Particle and Droplet

In dental settings, particles are generated from patient–provider interactions, dental equipment, and dental procedures. Our review defines droplets as water-based with a mean diameter >5 μm. Aerosols (or airborne nuclei) are defined as liquid or solid particles typically <5 μm in diameter. Particulate matters (PMs) are arguably interchangeable with aerosols and typically refer to solid (or liquid) particles in the size range of sub-micron to 10 μm. Particle concentration refers to the count (or particle mass) per unit volume, e.g., count/m^3^ (or μg/L).

Size influences the behavior and trajectory of droplets and aerosols/particles. Within a 1 m radius, droplets larger than 50 μm display ballistic or jet-like movements. Intermediate size droplets can either fall on the surface or can stay in the air and travel approximately 2 m before settling down. Smaller droplets and aerosols are the least impacted by gravity and can stay airborne for long durations. For example, a dust particle with a diameter of 10 μm falls 1 m in air at rest in ~5.5 min, whereas a 1 μm particle takes over 9 h to travel the same distance. Owing to air currents, such small particles may spread throughout the room, especially after the volatile liquid in the droplet dries out (17).

### Bioaerosols Production and the Influencing Factors

Human lungs inspire approximately 0.5–0.75 L per breath during rest. Air expelled through nasal respiration produces an exit velocity of ~0.5 m/s, whereas the exit velocity while speaking with a normal volume and pace exhibits ~0.3 m/s. In contrast, during periodic coughing, the exit velocity dramatically increases to 4–5 m/s (18). Direct observations of human sneezing and coughing reveal that these airflows consist of initially hot and moist turbulence followed by cool and buoyant clouds containing droplets of varying sizes. A single cough may expel ~700 particles as compared with a sneeze that may produce over 40,000 particles (19). In this regard, most of the particles and droplets produced from a sneeze are relatively large and may be easily blocked by the use of a simple mask. In addition to coughing and sneezing, speaking can generate droplets of size ranging from 20 to 500 μm (5). However, the estimated concentration of droplets per cough is 2.4–5.2 cm^−3^, which is significantly more than that of speech, 0.004–0.223 cm^−3^ (20).

In addition to particle size, environmental factors such as temperature and relative humidity influence the potency and distribution of infectious respiratory droplets and particles. For example, a 100 μm droplet will evaporate in approximately 10 s after expulsion while a 1 μm droplet will evaporate within 0.001 s (19). Increased air temperature, however, leads to an immediate decrease in particles post expulsion. By contrast, however, in an environment of elevated humidity, these numbers increase. Regardless of humidity or temperature, particles or droplets with a diameter of <0.1 μm evaporate almost immediately or cannot contain enough viral material to be infectious (9).

Viruses can be transmitted *via* droplets produced by sneezing and coughing, with diameters varying in the range of 0.1 μm–0.9 mm (21). Once an individual begins coughing, the duration and position of the mouth influence the area covered by the expelled droplets, which can degrade into categories of smaller size (9). Viral, fungal, and bacterial particles react in different ways depending on changes in temperature or humidity of the environment. The maximum stability of influenza occurs at 20–40% humidity and has a minimum stability at 50% humidity (22). Studies have suggested that SARS-CoV-2 exhibits similar survival curves in response to increases in temperature and humidity (23). With respect to coronaviruses at room temperature, they remain viable on surfaces for up to 9 days (24). At temperatures >30°C, the survival of these viruses decreases dramatically (24).

### Bioaerosols in the Practice of Dentistry

#### Dissemination of Microorganisms

Dissemination of microorganisms in dental operatories can occur directly, by contact with bacteria on the surfaces of dental instruments and dental providers, or indirectly, *via* splatter of droplets larger than 100 μm in diameter or by particles <100 μm in diameter suspended in air (25). Most dental bioaerosol studies have investigated bacterial colonies on the surfaces of dental instruments as the main pathogen. The potential for disease transmission of airborne bacteria (e.g., tuberculosis), viruses (e.g., measles and SARS), and bloodborne viruses, which can become aerosolized by blood splatter *via* high-speed handpieces used in dentistry and orthodontics, is not known (26–28). COVID-19 transmission is primarily through respiratory droplets and less likely through fomite transmission (29). The two most notable sources of droplet and aerosol generation in dental settings are procedures involving air turbine handpieces and ultrasonic instruments.

#### Air Turbine Handpieces

Studies have demonstrated that air turbine handpieces atomize 20 times more bacteria when compared to air spray. This production of bioaerosols is equivalent to the concentration produced by biological motions such as sneezing (30). A prophylactic hygiene handpiece with a pumice cup and pumice is often used for cleaning teeth. This common dental procedure produces a volume of aerosolized bacteria comparable to that resulting from a cough (31). *Tubercle bacilli* were found in droplet scatterings generated by dental air turbine handpieces within a range of 6 inches to over 4 feet from the patient’s mouth (26). This distance is larger than the distance between providers and patients, indicating a working dentist/assistant will undoubtedly be affected (30).

#### Ultrasonic Instruments

Ultrasonic instruments also produce significant amounts of aerosols and the vibration of the tip generates significant amounts of heat. Water is used to cool the instrument, which results in the generation of significant splatter. When mixed with saliva and plaque from the oral cavity, aerosolized splatter from ultrasonic instruments has the potential to become highly infectious and a major risk factor for disease transmission (31). Moreover, ultrasonic instrumentation can transmit 100,000 microbes/ft^3^ with aerosolization of up to 6 ft (32). In the absence of a favorable air current, microbes can survive for a period ranging from 35 min to 17 h. Using microbiological analysis, significantly higher bacterial counts were detected after scaling treatments, with the presence of Staphylococcus and Streptococcus species being the most notable (26). Results also showed high numbers of colony forming units (CFUs) and identified strictly oral anaerobes on all microbial plates from both groups, which meant that a significant amount of contamination occurred during ultrasonic scaling (26).

## METHODS OF CHARACTERIZING BIOAEROSOLS

Aerosolized particles can be classified into three categories: natural (e.g., fog, dust, and mist), anthropogenic (e.g., air pollution and smoke), and biological (e.g., bioaerosols) which are primarily released by humans and animals. These particles are carried through natural and anthropogenic means (33, 34). Bioaerosols contain both volatile and non-volatile material and their behavior and transmissibility depend on their size. Smaller bioaerosol particles penetrate more easily and go farther into the respiratory tract, which means they are more likely to transmit diseases compared to the larger particles (19).

**FIGURE 1 |:**
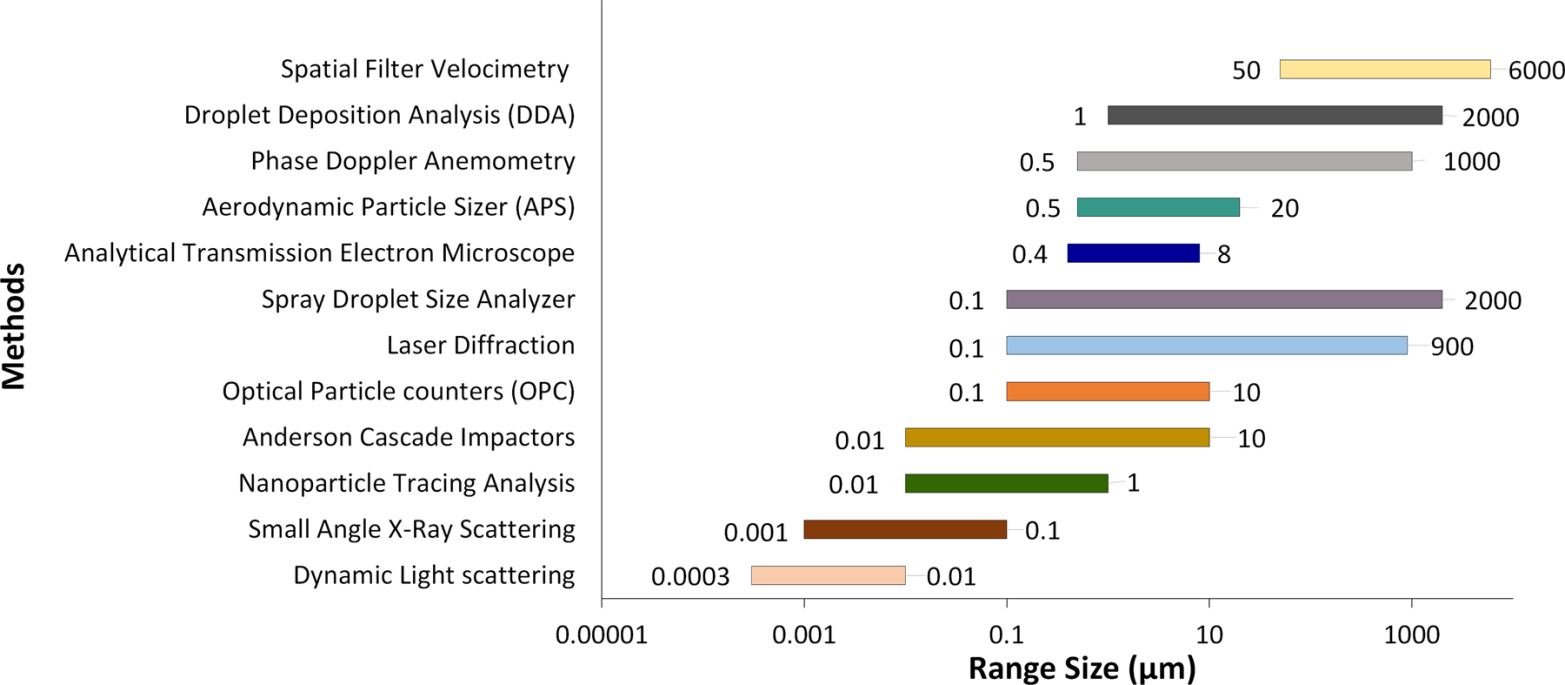
A summary of identified bioaerosol particle size distributions measured using select methods.

Over the years, researchers have developed a variety of instruments to assist with particle sizing and classification. A broad list of instruments used in bioaerosol particle counting and sizing is presented in Table 1, and a summary of measured particle size distributions is shown in Figure 1. More recently, researchers have started using machine learning to analyze SARS-CoV-2 infected particles (44).

## RECOMMENDATIONS TO MINIMIZE COVID-19 TRANSMISSION IN DENTAL SETTINGS

### Patient/Staff as Source of Particle

Although there are differences in aerodynamic behavior and properties between droplets and particles, both provide mechanisms for transmitting pathogenic microorganisms between patient and dental personnel. Human interactions (speaking, sneezing, and coughing), even without any symptom, can be a source of respiratory pathogen transmission in an indoor setting (5, 20), such as in dental offices. Dental providers working with high-speed handpieces have routine exposure to bodily fluids including respiratory particles and oral secretions (10, 34). Ultrasonic scalers, air turbines, three-in-one syringes, and air-water syringes are also significant contributors to bioaerosol generation (31).

#### Personal Protective Equipment

Personal protective equipment (PPE) is an important mitigation strategy (45). Global sources for producing PPE continue to be insufficient due to a large number of COVID-19 cases, misinformation, panic buying, and stockpiling (6). It is imperative to revisit the current and developing PPE options with respect to their efficacy. The WHO has listed the following PPEs as necessary for healthcare workers: medical masks, N95 respirators, filtering facepiece respirators-2 (FFP2) standard or equivalent, gowns, gloves, aprons, and eye protection (goggles or face shields) (6). A recent study on COVID-19 prevalence among dentists, while adhering to the listed PPEs, indicated a very low percentage of only 0.9% (16). While combinations of PPE are recommended, the effectiveness of specific PPE at preventing SARS-CoV-2 infection has not been quantified. Determining the effectiveness of PPE is complicated due to the limited controlled human infection studies. “Silent spreaders” may expose healthcare workers at work and elsewhere if basic non-pharmaceutical interventions are not universally adopted and enforced (46, 47). In the absence of PPE measures, historical data from similarly transmitted respiratory diseases, such as tuberculosis in dental settings showed a transmission of 10%, which was more than double the reported data from the National Health and Nutrition Examination Survey (NHANES) in the year 2000 (48).

#### Face Shields

Most harmful particles are generated during the initial part of a cough (49). Healthcare providers working at a distance of 46 cm from an infected patient may inhale 0.9% of these harmful particles. Wearing a face shield can reduce the inhalation of aerosols by 96% and surface contamination by 97% for droplets with a volume median diameter (VMD) of 8.5 μm. However, for smaller droplets with a VMD of 3.4 μm, the reduction rates are lower with 68 and 76% for aerosols and surface contamination, respectively (45). Face shields may reduce the inhalation of large harmful aerosol particles for a short time. However, smaller particles remain in the air for longer periods and could bypass the face shield. These particles pose inhalation risks for healthcare providers and patients (50).

#### Face Masks

A study conducted on 47 human subjects with influenza showed that the average cough profiles had a volume of 4.16 L with a peak flow rate of 11.1 L/s (22). A mask designed to suppress droplets at this volume and flow rate could be an effective inhibitor for smaller cough volumes and lesser peak flow rates (18). A different study reported that each patient suffering from influenza released ~38 pL of particles in the size range of <10 μm. After being diagnosed and receiving treatment, the study participants released ~26 pL of particles per cough. Droplets become infectious when they encounter the mucous membranes, e.g., oral and nasal cavities of the body (51). Studies on influenza-related diseases support the use of surgical masks for effective reduction of infection (47). Using masks in crowded places could reduce the risk of contracting influenza-like respiratory infections by 20% (52). Masks made of foam, cloth, or paper are less effective at filtering bacterial aerosols (50).

#### Masks vs. Respirators

According to the CDC, the N95 FFP can block at least 95% of 0.3 μm particles. Most research pertaining to the efficacy of face masks has been done by quantifying the number of respiratory viruses in exhaled breaths of participants with acute respiratory virus illness (17). Surgical masks can effectively reduce the emission of influenza-laden respiratory droplets but not particles (53). When subjects cough while wearing a surgical mask or N95, the dispersion of forward moving viral aerosol particles decreases but the lateral dispersion patterns of particles increases (54). N95s and surgical masks offer similar levels of protection against viral infection of respiratory diseases in non-aerosol producing environments (55).

### Equipment as Source of Particles (Room Design/Equipment Modification)

Dental buildings can contain high levels of circulating bioaerosols. Air-conditioning and ventilation systems in these settings should be maintained on a regular basis to minimize recirculation of contaminants (56). Cooling towers, air-conditioning, and mechanical ventilation systems are known sources of *Legionella pneumophila* (57). The risks to dentists, patients, and others who are routinely exposed to bioaerosols remain unclear, prompting the need for further research (14).

#### Office or Clinic Design

Multiple actions can be taken to decrease the transmission of infectious particles in healthcare settings. Electronic-based patient triages and check-ins, automatic doors, motion-sensing lights, and hand-sanitizer dispensers reduce the physical interface among patients, physicians, and interior surfaces. The use of thermal imaging to screen for elevated body temperatures ensures a safe distance between ingress patients and office staff while shortening the initial screening process. Since dental procedures generate mists and aerosols, local exhaust ventilation should be positioned with consideration of the aerosol flow direction as well as the location of the physician and the patient (9, 58–61).

#### Heating, Ventilation, and Air Conditioning System Design

Dental offices are advised to use a systems-based approach with engineering controls to minimize cross contamination. For example, fans could be used to direct the airflow first through the staff working areas and then through the patient treatment areas, thereby reducing workplace risks involving airborne particles or droplets (62). Positioning of patients in front of each other should be avoided whenever possible (9). Operatories should be oriented parallel to the direction of airflow, which will assist in directing the flow of airborne contaminants (62).

The CDC also recommends positioning the heads of patients away from pedestrian corridors and closer to the back wall and return air vents (9, 63). Several studies have evaluated the characteristics of plumes generated by exhaled droplets and noted that a top exhaust system is more efficient than the traditional air conditioning systems (64). Ultraviolet (UV) germicidal irradiation, including UV-C at 254 nm, in conjunction with ventilation is emerging as a cost-effective tool for reducing viral aerosols (52, 65, 66). In the design of future dental offices, the designation of negative-pressure isolation rooms, antechambers, and 24/7 HVAC systems could improve aerobiological controls (63).

#### Interior Design

It is important to consider an interior design that prompts safe conduct of ordinary activities. Visual signage is a good option to communicate instructions clearly to the general public. For example, clear marking of risk zones with visual aids or creating visual cues for specific activities raises awareness and allows policies to be followed easily (58). Additionally, creating signage that indicates “clean” areas around donning rooms and PPE carts can establish easy-to-follow protocols. Efficient and regimented routines create less interaction and reduce cross-contamination. The efficacy of visual signage is increased if supplemented with an office culture based on safety and education. Additionally, anti-bacterial surface coatings reduce healthcare-associated infections (HAIs) by 36% while also decreasing CFUs and clinically relevant pathogens by ~59–75% (67). Other design initiatives worth considering are sanitizing stations, maintaining social distancing whenever possible in the reception and treatment rooms, and dedicated PPE recycling bins in each room (58).

## CONCLUSIONS

Respiratory droplets are the primary mode of SARS-CoV-2 transmission. Human and simulated studies have demonstrated that sneezing and high-volume coughs in patients pose a significant risk for viral transmission. Environmental conditions effect the potency of viruses shed from human coughing and sneezing as well as dental procedures. SARS-CoV-2 reacts inversely to temperature and humidity, which are factors that can be controlled in a closed dental setting. One of the biggest concerns for dental providers is the working proximity to potentially active SARS-CoV-2 sources, as intermediate size (10–50 μm) droplets can travel as far as 2 m away from the source. Furthermore, these droplets are easily disseminated by air currents. Surgical masks and respirators appear to provide significant protection against viral particle transmission from infected individuals, although the risk is not completely eliminated. While we highlighted various methods used to study the size and distribution of airborne droplets and particles, these methods for the most part do not measure the viral load and more specialized tools need to be developed. Implementing some of the recommendations we proposed and a greater awareness amongst dental practitioners about the relationship between particle dynamics and clinical dentistry, can help improve safety in dental practice. Finally, while this review provides a broad overview of past and current studies related to pathogen and particle transmission in dental settings, there is a clear gap in our understanding of how dental practices specifically affect the transmission of SARS-CoV-2; therefore, much more research is needed in this area. This knowledge is imperative to addressing the current crisis, and those that might be faced in the future.

## Figures and Tables

**TABLE 1 |:** Experimental methods used for particle count and characterization.

Method	Description	Select bioaerosol studies and comments
Aerodynamic particle sizer (APS)	Uses the principle of inertia to size particles. Particles pass between two laser beams and the scattered light is collected on a photodetector. By measuring the time delay between pulses generated as particles pass through the laser beams, the velocity and diameter of particles are measured.	Morawska et al. (35): studied particle concentration and size distribution near the mouth for a range of breath exercises. Voiced activities produced higher particle concentrations than whispered ones which indicated particles were produced by the vibrating vocal cords. Whispered counting and breathing produced similar amount of particles.
Andersen cascade impactors (ACI)	Also known as cascade sampler impactors. Used to measure the size distribution of non-volatile aerosolized particles (36). A suction pump is used to draw air through a series of 6–8 stages which are used to separate different particle sizes.	Two types can be found; one for viable particles (meaning viruses and bacteria which can be grown on a series of Petri dishes) and the other for non-viable particles.
Droplet deposition analysis (DDA)	Uses optical or electron microscopes to measure the size of deposited droplets on a surface by using a substrate which preserves traces of the deposited droplets.	Duguid (37): measured the droplet size of sneezing, coughing, and speaking. Found similar size distribution for all activities, but smaller droplets much more frequent in sneezing. 95% of droplets were between 2 and 100 μm. Most common are in the range of 4–8 μm (38).
Interferometric Mie imaging (IMI) and particle image velocimetry (PIV)	An out-of-focus imaging technique of particles illuminated by a laser light sheet (39). It may be used simultaneously with particle image/tracking velocimetry (PIV/PTV) to measure instantaneous velocity fields.	VanSciver et al. (40): measured cough velocity of 29 healthy subjects within the range of 1.5 and 28.8 m/s. Chao et al. (20): studied 11 human subjects and measured the size distribution and velocity of droplets during speaking and coughing using IMI APS and PIV. Found the mean diameter of particles was 13.5 and 16 μm and velocity was 11.7 and 3.1 m/s for coughing and speaking respectively. Zhu et al. (41): studied transport properties of the saliva droplets of coughing in an indoor environment by using both PIV and numerical methods. The initial coughing velocity was estimated between 6 and 22 m/s with an average velocity of 11.2 m/s and the impacted area was 2 m or larger.
Laser diffraction (LD)	Utilizes the light scattering principle to measure the distribution of particle size by determining the unique variations in the intensity of light scattered as a laser beam travels through a scattered particulate sample. Large particles scatter light at small angles and vice versa. The angular light intensity data is then evaluated to assess the size of the particles responsible for producing such scattering patterns.	Zayas et al. (21): used laser diffraction to measure voluntary cough aerosols of 45 healthy non-smokers and found a size range of 0.1–900 μm of which 97% of droplets were found to be less than 1 μm.
Optical particle counters (OPC)	Works on the concept of light scattering from illuminated particles. Two types are generally found: LED and laser-based counters; the first is better for counting larger particles, while the latter is better for smaller particles.	Papineni and Rosenthal (42): measured exhaled droplets from mouth breathing, nose breathing, coughing, and talking. They also used an analytical transmission electron microscope (analytical TEM) and found particle sizes with the OPC in the range of 0.3–2.5 μm and with the analytical TEM in the range of 0.4–7.6 μm. Edwards et al. (43): measured expired air particle count and size and reported the size range of droplets between 0.085 μm and >0.5 μm with a mode between 0.15 and 0.2 μm.
Spray droplet size analyzer (SDSA)	A laser diffraction-based droplet sizer that can detect aerosols and particles between 0.1–2,000 μm	Lindsley et al. (22): measured the concentration of aerosols from a cough aerosol simulator. A major peak in aerosol concentrations was measured in the size range of 3–10 μm.
